# The impact of positive and negative affect on aperiodic EEG activity: evidence for a shared neural basis of metacontrol and emotion

**DOI:** 10.1093/cercor/bhaf335

**Published:** 2026-01-07

**Authors:** Jing Fan, Shuhui Lyu, Xiaolei Xu, Lorenza Colzato, Bernhard Hommel

**Affiliations:** Faculty of Psychology, Shandong Normal University, No. 88 East Wenhua Road, Jinan 250014, China; Shandong Provincial Key Laboratory of Brain Science and Mental Health, No. 88 East Wenhua Road, Jinan 250014, China; Faculty of Psychology, Shandong Normal University, No. 88 East Wenhua Road, Jinan 250014, China; Shandong Provincial Key Laboratory of Brain Science and Mental Health, No. 88 East Wenhua Road, Jinan 250014, China; Faculty of Psychology, Shandong Normal University, No. 88 East Wenhua Road, Jinan 250014, China; Shandong Provincial Key Laboratory of Brain Science and Mental Health, No. 88 East Wenhua Road, Jinan 250014, China; Faculty of Psychology, Shandong Normal University, No. 88 East Wenhua Road, Jinan 250014, China; Shandong Provincial Key Laboratory of Brain Science and Mental Health, No. 88 East Wenhua Road, Jinan 250014, China; Faculty of Psychology, Shandong Normal University, No. 88 East Wenhua Road, Jinan 250014, China; Shandong Provincial Key Laboratory of Brain Science and Mental Health, No. 88 East Wenhua Road, Jinan 250014, China

**Keywords:** EEG aperiodic activity, emotion, cognitive control, metacontrol, affect

## Abstract

Metacontrol refers to the ability to dynamically adjust cognitive-control strategies, ensuring a balance between persistence and flexibility. Empirical findings point to a strong link between metacontrol and emotion, but the mechanistic underpinnings of this link remain unknown. Here, we had two goals. First, we hypothesized that metacontrol and emotion are mechanistically linked through aperiodic EEG activity, in the sense that both positive emotion and metacontrol flexibility come with increases, and both negative emotion and metacontrol persistence with decreases of aperiodic activity. Second, we tested whether and to what degree emotional stimuli affect behavior and aperiodic activity automatically. In a large sample (*n* = 120), we examined EEG and behavioral data from three tasks in which we systematically varied the task-relevance of the emotional information presented to participants. As hypothesized, positive pictures resulted in higher aperiodic activity than negative pictures. Task context and, more specifically, the relevance of emotional stimuli significantly influenced overt behavior but had no effect on aperiodic activity. We conclude that positive and negative emotions may represent the phenomenal “feel” of metacontrol biases towards flexibility and persistence, respectively, and that the degree to which processes are affected by emotional content automatically depends on the process under consideration.

## Introduction

To account for the human ability to orchestrate cognitive functions in task- and goal-specific ways, researchers often refer to the concept of cognitive control. However, traditional control approaches (eg Miller and Cohen 2001) have commonly focused on tasks that require a high degree of selectivity and concentration, like in a Stroop or flanker task (Stroop 1935; Erikse and Eriksen 1974), where one single aspect of a stimulus display is relevant but everything else needs to be ignored. However, more recent approaches suggest that this kind of control represents only one side of the control coin. While a high degree of selectivity and focus is indeed important in many tasks used by cognitive researchers and many situations people face in real life, other tasks and situations call for the exact opposite, namely, for changing rather than sticking to one’s goal, to open up the system for alternatives, and to integrate as much information as possible. Accordingly, some researchers (Cools et al. 2008; Durstewitz and Seamans 2008; Goschke and Bolte 2014; Hommel and Colzato 2017) have suggested that true human adaptivity requires the balancing between a control style that establishes a high degree of selectivity and focus, often called persistence, and a control style that establishes a high degree of openness and integration, often called flexibility. Hommel and colleagues have coined the dynamic regulation of cognitive control styles to achieve an optimal balance between persistence and flexibility metacontrol (Hommel 2015; Hommel and Colzato 2017; Hommel and Wiers, 2017; Hommel et al. 2024).

Of particular importance for the present study are theoretical assumptions and empirical findings suggesting a strong link between metacontrol and emotion. Dreisbach and Goschke (2004) and Hommel and Colzato (2017) have suggested and provided evidence that positive emotion might be associated with a metacontrol bias towards flexibility, whereas the opposite might be true for negative emotion. Indeed, positive mood has been found to facilitate brainstorming for new solutions (Nijstad et al. 2010), increase distractibility (Dreisbach and Goschke 2004), and hamper the fine-tuning of persistence to avoid the processing of distractors (van Steenbergen et al. 2010), whereas negative mood was found to reduce crosstalk between tasks (Zwosta et al. 2013). Conversely, engaging in a task that calls for flexibility was reported to result in positive-going mood, whereas tasks that call for persistence tended to result in more negative mood (Akbari Chermahini and Hommel 2012). This suggests that metacontrol and emotion do indeed have a lot in common. The question is, however, why that should be the case.

Earlier approaches (Dreisbach and Goschke 2004; Dreisbach 2006) considered emotional states a cause and changes in cognitive control style the effect, but it remained unclear why and based on which mechanism having a particular emotion should have an impact on how one exerts cognitive control. In contrast, Hommel (2019, 2023, 2025) considered the conceptual overlap of emotion on the one hand and control on the other, and argued that both may share a substantial degree of cognitive and neural mechanisms. In other words, emotion and control may in some sense be the same thing. Indeed, human emotion is assumed to rely on dopaminergic and serotonergic activity, especially in the striatum (Dayan and Huys 2009; Eshel et al. 2024), which fits with the assumption that metacontrol emerges from the interplay between frontal and striatal dopaminergic pathways (Cools et al. 2008; Durstewitz and Seamans 2008; Hommel and Colzato 2017), which in turn are moderated by serotonin (Daw et al. 2002; Di Giovanni et al. 2008). Hence, the idea that metacontrol shifts and emotional feelings are generated by the same neurochemical machinery, at least to some degree, is consistent with basic assumptions in emotion and metacontrol research. In other words, positive and negative emotion might represent how biases towards flexibility and persistence feel (Hommel 2019).

Motivated by these insights, the present study aimed to get a better understanding of the functional and neural underpinnings of the mechanisms that emotion and control might share. As elaborated by Hommel and Colzato (2017), metacontrol persistence might emerge from a particularly strong impact of the present goal on decision-making, together with a strong mutual competition between decision-making alternatives, whereas metacontrol flexibility might emerge from the opposite. If so, fewer options and choices should be activated under a persistence bias as compared to a flexibility bias. In other words, the cognitive system/the brain can be expected to be “noisier” if optimized for flexibility than for persistence. From this angle, it is particularly interesting that metacontrol biases towards persistence and flexibility were found to be systematically related to neural measures of cortical noise as assessed by the FOOOF (Fitting Oscillations & One-Over-F) algorithm developed by Donoghue et al. (2020). The FOOOF exponent that this algorithm calculates captures the degree to which EEG signals display aperiodic activity, that is, the activity that remains after extracting all the activity captured by the standard EEG frequency bands (alpha, beta, delta, gamma, theta). Aperiodic fluctuations in neural signals serve as a reliable indicator of the excitation/inhibition (E/I) equilibrium within the cortical network (Gao et al. 2017), with alterations in the aperiodic exponent reflecting shifts in the balance between inhibitory and excitatory processes. An elevated aperiodic exponent signifies predominant inhibitory activity, reinforcing cortical stability and enhancing neural signal fidelity, whereas a reduced exponent indicates heightened excitatory dynamics, contributing to greater cortical noise and diminished efficiency in information transmission (Lombardi et al. 2017). Specifically, a lower aperiodic exponent correlates with increased cortical instability and neural interference, while a higher exponent suggests a more structured and top-down controlled neural state, optimizing cognitive functioning (Voytek and Knight 2015).

Of particular interest for our purposes, task conditions that call for a high degree of persistence were found to be associated with a relatively high value of the exponent, indicating little noise/dominance of inhibition, while task conditions that call for a high degree of flexibility were found to be associated with relatively low values of the exponent, indicating more noise/dominance of excitation (Zhang et al. 2023; Gao et al. 2024, 2025; Pi et al. 2024, 2025; Yan et al. 2024, 2025). Applied to the assumed relationship between metacontrol and emotion, this implies that positive-going affect should come with a lower aperiodic exponent than negative-going affect. While the relationship between affective changes and aperiodic activity has not yet been studied, a recent reanalysis of data from a memory study provided findings consistent with our general expectation (Xu et al. 2025). These authors reported that the frequent and consistent presentation of happy faces during a block of an n-back task, where participants were to decide whether the present face would show the same person that appeared two trials before, yielded a lower aperiodic exponent than the frequent and consistent presentation of sad faces. Notably, this effect was not found when the valence of the faces varied randomly from trial to trial (suggesting an effect of mood), but this may have been due to the rather complicated structure of the task and the need to inhibit the immediately preceding stimulus to solve it. In any case, we aimed to test the impact of brief affective states on aperiodic activity in a simpler task and in the absence of any ongoing memory requirement. Accordingly, we presented participants with pictures that are known to evoke positive and negative affect, and we predicted that positive pictures result in a lower aperiodic exponent, thus indicating more cortical noise, than negative pictures. This pattern would support the assumption that positive affect and flexibility biases in metacontrol are sharing a noisier and more excitation-dominated neural state, whereas negative affect and persistence biases are sharing a less noisy and more inhibition-dominated neural state.

In addition to this first aim of our study, we also had a second aim that was motivated by the ongoing controversy in emotion research regarding the automaticity of the effects of emotional stimuli. Even though many theoretical approaches to emotion assume that affective valence exerts automatic effects on cognition, discrepancies in empirical findings suggest that the extent of automaticity may depend on task relevance and contextual factors (Pessoa 2017). Whether, and to which degree this is the case, we tested by systematically varying the task-relevance of the emotional information that we presented to participants. In the first of our three tasks (Task 1), which varied within participants, the emotional stimulus (ie the picture with positive or negative content) was entirely irrelevant to the task, presented as a “prime” that preceded the actual stimulus, and participants could not take any benefit from processing the emotional prime. Approaches that assume complete automaticity of the processing of affective valence (Zajonc 1980; Barrett et al. 2007) would nevertheless predict full processing of the emotional content which, according to our first hypothesis, should result in the same kind and degree of impact on the aperiodic exponent than in the other two tasks, where the emotional stimulus was not entirely irrelevant. In Task 2, the emotional stimulus was integrated with the task-relevant stimulus both temporally and spatially, so that attending to the relevant stimulus also enforced attending the emotional stimulus to some degree. Even though the content of the emotional stimulus was unrelated to the task and to the relevant stimulus, the spatio-temporal characteristics of the irrelevant stimulus did provide information about the relevant stimulus, thus introducing some degree of task-relevance. In Task 3, the valence of the emotional stimulus provided the task-relevant information, so that processing the stimulus was essential for performance. If the impact of valence on the aperiodic exponent would be the same for all three tasks, this would provide strong evidence for a full-automaticity approach, whereas a strong effect in Task 3 but increasingly smaller or absent effects in Task 2 and Task 1 would be more consistent with claims of conditional control of the processing of emotional stimuli.

In addition to our primary analyses, we also explored whether stable personality traits, as captured by the Big Five model, are related to individual variability in aperiodic EEG activity and emotional processing. Personality dimensions have been linked to cortical E/I dynamics and affective reactivity (Pacheco et al. 2024). Including these exploratory analyses allowed us to examine whether inter-individual differences in personality contribute to the variability of neurophysiological responses observed across tasks.

## Materials and methods

### Participants

A power analysis was conducted using G*Power 3.1 (Faul et al. 2007) to determine the required sample size. Based on an effect size of *f* = 0.25 (medium effect), α = 0.05, and power = 0.90 for detecting within-subject effects in our repeated measures design, the analysis indicated that 120 participants would provide sufficient statistical power. Accordingly, we recruited 120 right-handed healthy young adults (60 males, Mage = 20.90 ± 1.55 yr; 60 females, Mage = 20.60 ± 1.18 yr) from Shandong Normal University, Qilu University of Technology and Shandong University in Jinan, China. All participants were aged between 18 and 30 yr and had normal or corrected-to-normal vision. Before participating, all individuals gave written informed consent after being thoroughly informed about the experimental procedures. Participants received monetary compensation (100 RMB) upon completion of the study. The research protocol (ID: SDNU2024062) was approved by the Ethics Committee of the School of Psychology at Shandong Normal University and conducted in accordance with the Declaration of Helsinki.

### Research design

The within-subject cross-sectional study was carried out in an EEG laboratory at the Metacontrol Lab of Shandong Normal University’s School of Psychology. The participants completed the BIG 5 questionnaire at the beginning and then commenced with a resting-state EEG measurement, followed by the three cognitive tasks, carried out in fully balanced order, alongside EEG recording.

### Personality questionnaire

As an exploratory component of our study, the BIG 5 personality traits were assessed using The Chinese Big Five Personality Inventory (Wang et al. 2011), brief version, a validated 40 items questionnaire, was employed to assess participants’ personality traits across five core dimensions: (i) neuroticism; (ii) conscientiousness; (iii) agreeableness; (iv) openness; and (v) extraversion. Each domain comprises 8 items rated on a 6-point Likert scale (1 = Strongly disagree to 6 = Strongly agree). Seven reverse-coded items were recalculated before computing dimension scores which ranges from 8 to 40, with higher values indicating stronger trait expression. The CBF extr subscales demonstrated strong reliability and validity in our sample with all Cronbach’s α ≥ 0.763 and KMO ≥ 0.743 in all scales, and significant Bartlett’s test of sphericity (all *P* < 0.001), confirming their suitability for subsequent analyses.

### Resting-state EEG

The open-eye resting EEG activity of participants was recorded for 5 min using 64 Ag/AgCl electrodes arranged isometrically, while a fixation cross remained visible on the screen.

### Experimental paradigm

**Fig. 1:**
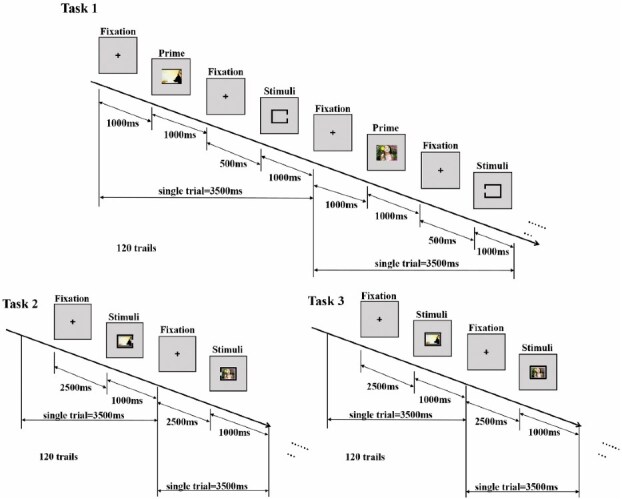
The frames and pictures paradigm involved three tasks. In Task 1 and Task 2, emotional stimuli were irrelevant to the task, with participants focusing on judging the gap direction of a black frame—while ignoring distractors (Task 1) and then while they appeared alongside the target (Task 2). In Task 3, emotional stimuli became relevant, requiring participants to classify picture valence while still assessing the frame’s gap direction. In all three tasks, each single trial took 3,500 ms.

We developed a novel “Frames and Pictures” paradigm, inspired by Xu et al. (2024), and implemented it using E-Prime 2.0 (Psychology Software Tools, Pittsburgh, PA). As illustrated in Fig. 1, the paradigm was designed to investigate how emotional relevance modulates attentional and neurophysiological processing. To this end, it included three tasks that systematically varied the degree to which emotional pictures were relevant to the participant’s behavioral goal. Across all tasks, participants viewed a series of pictures drawn from the International Affective Picture System (IAPS; Lang et al. 2008), comprising 30 positive and 30 negative images that differed significantly in standardized valence ratings.

The three tasks were identical in basic structure—each involving the presentation of a black frame and an emotional picture—but differed in whether the emotional stimulus was task-relevant or task-irrelevant. In Task 1, emotional pictures were presented outside the frame, functioning as distractors unrelated to the participant’s goal. Participants were instructed to ignore these pictures and focus solely on judging the gap direction (left or right) of the black frame. In Task 2, emotional pictures were still task-irrelevant but appeared inside the frame simultaneously with the target, thereby increasing their perceptual and attentional salience while the task instructions remained the same (gap-direction judgment). Finally, in Task 3, the emotional pictures became fully task-relevant. Here, participants were asked to classify the valence of the image (positive or negative) presented within the frame, directly engaging emotion processing.

Each task consisted of 120 trials (60 positive and 60 negative), with stimulus timing adjusted according to task demands—whether the emotional image appeared preceding, concurrent with, or as the main target. Participants responded using the “F” and “J” keys on the keyboard, indicating either gap direction (Tasks 1 and 2) or emotional valence (Task 3). They were instructed to respond as quickly and accurately as possible while maintaining central fixation throughout all tasks. Before the main experiment, participants completed a short practice session to ensure that task instructions and response mappings were fully understood.

The complete experimental session, including three tasks and practice trials, lasted approximately 25 min per participant—an interval considered too brief to induce substantial cognitive or attentional fatigue in healthy adults (Ackerman and Kanfer 2009). This design allowed us to manipulate the behavioral relevance of emotional information in a controlled, parametric way—progressing from complete irrelevance (Task 1) to full relevance (Task 3)—while maintaining comparable visual input and motor output demands. This structure also ensured that differences across tasks could be attributed to the changing emotional salience rather than to general task difficulty or perceptual differences.

### E‌EG recording and analysis

A total of 64 equidistant Ag/AgCl electrodes, facilitated by QuickAmp and BrainAmp amplifiers (Brain Products GmbH, Gilching, Germany) were used to record the EEG activity throughout the experiment. The ground electrode was located at spherical coordinates (θ = 58°, φ = 78°), with the reference electrode positioned at (θ = 90°, φ = 90°). All recordings were obtained at a sampling frequency of 500 Hz, with electrode impedances kept below 10 kΩ.

The data were preprocessed offline using Analyzer 3.0. Before any following analysis, bad channels were identified and interpolated. The EEG signals were subsequently re-referenced to average reference, followed by band-pass filtering between 0.1 and 45 Hz using an eighth-order infinite impulse response filter, with additional 50 Hz notch filter. Afterwards, the EEG data were processed using Independent Component Analysis with the Infomax restricted algorithm to identify and manually eliminate stereotypical artifacts including ocular (horizontal/vertical eye movements and blinks) and cardiac interference. Following this, additional semi-automatic inspection (trials containing: (i) extreme amplitudes, ±100 μV threshold, or (ii) flatline periods, < 0.5 μV changes over 100 ms were reject automatically) was performed to ensure complete artifact removal.

The data were then epoch-locked to the emotional stimuli onset with each segmented trial comprising a 5,000 ms window, spanning from −2,000 ms pre-onset to +3,000 ms post-onset, and the segmentation included only trials with correct responses. At last, baseline correction was applied using the −200 ms to 0 ms interval (emotional pictures onset), followed by within-subjects averaging in all six conditions (3 task × 2 emotions).

### Parameterization of the spectral data

The time window spanning from −1,000 to 0 ms relative to stimulus onset was defined as the pretrial phase, whereas the 0 to 1,000 ms post stimulus interval constituted the within-trial phase. In order to calculate the aperiodic components, spectral decomposition was performed to extract aperiodic components by computing power spectral density (PSD). Using MATLAB’s “Welch” algorithm (Hamming window: 250 ms duration, 50% overlap; Welch 1967), we calculated PSD for all electrodes and participants for both pretrial and within-trial phases under each experimental condition.

A Python-based FOOOF toolbox (version 1.0.0; accessible at https://github.com/fooof-tools/fooof) was used to measure aperiodic activity. The FOOOF algorithm enables spectral decomposition by separating the signal into two distinct components: an aperiodic component (characterized by a 1/f slope) and periodic components (oscillations), thereby allowing for comprehensive parameterization of the power spectrum (Donoghue et al. 2020). The power spectrum model is formally expressed as:


$$ PSD(f)=L(f)+\sum_n{G}_n(f) $$

Formally, $L(f)$ represents the aperiodic component, and $\sum_n{G}_n(f)$ denotes the periodic component, where *f* represents frequency. The model decomposes the power spectrum into two distinct elements: (i) a linear combination of the aperiodic component represented by $L(f)$, and (ii) a set of oscillatory components modeled as n Gaussian functions. The aperiodic component, which spans the entire frequency range, is parameterized by two key variables that capture its spectral characteristics. The mathematical expression for the aperiodic component function $L(f)$ is given by:


$$ L(f)=b-\log \left[{f}^x\right] $$

As the model specifies, b represents the broadband power “offset” of the aperiodic component, *x* denotes the “exponent” of the aperiodic fit (corresponding to the slope of the power spectrum in log–log space), and *f* indicates frequency. The periodic components, also called oscillatory components, correspond to frequency bands where power exceeds the aperiodic component. Each oscillatory peak is modeled as a Gaussian function and is characterized by three parameters. The periodic component function ${G}_n(f)$ is expressed as:


$$ {G}_n(f)={a}_n\exp \left[-\frac{{\left(f-{\mu}_n\right)}^2}{2{\sigma}_n^2}\right] $$

This model parameterizes each oscillatory peak using three key features: ${a}_n$ for amplitude, ${\mu}_n$ for center frequency, and ${\sigma}_n$ for spectral bandwidth. Spectral fitting was performed within the 3 to 40 Hz range using constrained optimization parameters (peak_width_limits: 2 to 8 Hz; min_peak_height: 0.05; max_n_peaks: 8; aperiodic_mode: fixed). For each recording channel, subject, experimental condition, and time window, aperiodic exponent value were extracted through spectral decomposition. The spectral fit’s average R^2^ reached at least 0.89 across all 120 participants in all conditions, indicating excellent model convergence.

### Aperiodic exponent

The aperiodic signal is quantified by two physiologically meaningful parameters: (i) the exponent χ, capturing the 1/f slope, and (ii) the offset b, reflecting broadband power. Previous studies have given evidence of the validity of aperiodic exponent representing sensitivity of the metacontrol state (Zhang et al. 2023; Gao et al. 2024, 2025; Pi et al. 2024, 2025; Yan et al. 2024, 2025). Therefore, this study prioritized exponent as analysis target. For each participant, the aperiodic exponents of every electrode were extracted. The “global” exponent approach recommended by Hill et al. (2022) was adopted due to the absence of prior assumptions about the spatial distribution of aperiodic neural activity across the scalp. Eventually, the averaged aperiodic exponent values from all 64 electrodes of each participant were exported for further statistical analysis.

### Statistical analysis

All statistical analyses were performed using SPSS software (IBM, version 26.0). Outliers were defined as having fewer than 30 valid trials or having values exceeding ±3 standard deviations from the mean; they were removed and then replaced using missing value analysis conducted with the Expectation–Maximization algorithm (Amusa and Hossana 2024). Behavioral data (Percentage Errors and Reaction time, RT, trials with correct responses were exclusively selected) were analyzed separately using ANOVA with two within-subject factors: emotion type (positive vs. negative) and task (Task 1 vs. Task 2 vs. Task 3). Aperiodic exponents (whole-brain) were analyzed by means of three-way repeated measures ANOVAs, with time interval (pretrial period vs. within-trial period), emotion type (positive vs. negative) and task (Task 1 vs. Task 2 vs. Task 3) as within-subject factors. Separate aperiodic exponents were calculated for the interval before the stimulus was presented, as a local baseline, and the actual trial, starting with stimulus presentation, so to capture the actual task-specific cognitive work. Subsequently, a nonparametric cluster-based permutation test was utilized to identify significant electrodes in the theoretically relevant within-trial period. Two-way repeated measures ANOVA were then performed on identified clusters of electrodes. Further, to test whether the emotion effect can be predicted from the resting-state, we quantified the emotion effect by calculating Δ-emotion (Δ-Exponent = FOOOF value for negative pictures—FOOOF value for positive pictures, averaged across all three tasks in the within-trial phase; Δ-PE = percentage of error for negative pictures—percentage of error for positive pictures, averaged across all three tasks in the within-trial phase; Δ-RT = reaction time for negative pictures—reaction time for positive pictures, averaged across all three tasks in the within-trial phase), representing the individual-specific magnitude of emotional responses. Finally, a Pearson correlation analysis with Bonferroni correction for multiple tests was then conducted between BIG 5 scores, behavioral performance and FOOOF indices in both resting-state and task state, and Δ-emotion. Greenhouse–Geisser correction was used wherever suitable, and simple effects analyses were performed only when interactions reached significance. Post hoc tests were adjusted using the Bonferroni method. Descriptive statistics were provided as the mean along with the standard error of the mean (SEM).

## Results

### Behavior

#### Percentage of errors

For the behavioral performance (gap discrimination in Tasks 1 and 2, and emotion discrimination in Task 3), a two-way repeated-measures ANOVA (task × emotion) revealed significant main effects of task (*F*_[2238]_ = 282.75, *P* < 0.001, η2 *P* = 0.70) and emotion (*F*_[1119]_ = 73.26, *P* < 0.001, η2 *P* = 0.38), with Task 1 showing the lowest error rates (0.01 ± 0.00) and Task 3 the highest (0.13 ± 0.01); positive stimuli elicited lower error rates than negative stimuli (0.03 ± 0.00 vs. 0.07 ± 0.00, 95% CI = −0.043, −0.027). A significant task × emotion interaction was observed (*F*_[2238]_ = 64.80, *P* < 0.001, η2 *P* = 0.35). Post hoc Bonferroni-corrected analysis indicated that, while Task 1 showed consistently low error rates across emotional conditions (all *P* < 0.05), Task 3 exhibited consistently high error rates (all *P* < 0.001). Only Task 3 showed significant emotional modulation (positive vs. negative: *P* < 0.001, d = −0.10).

#### Reaction time

**Fig. 2:**
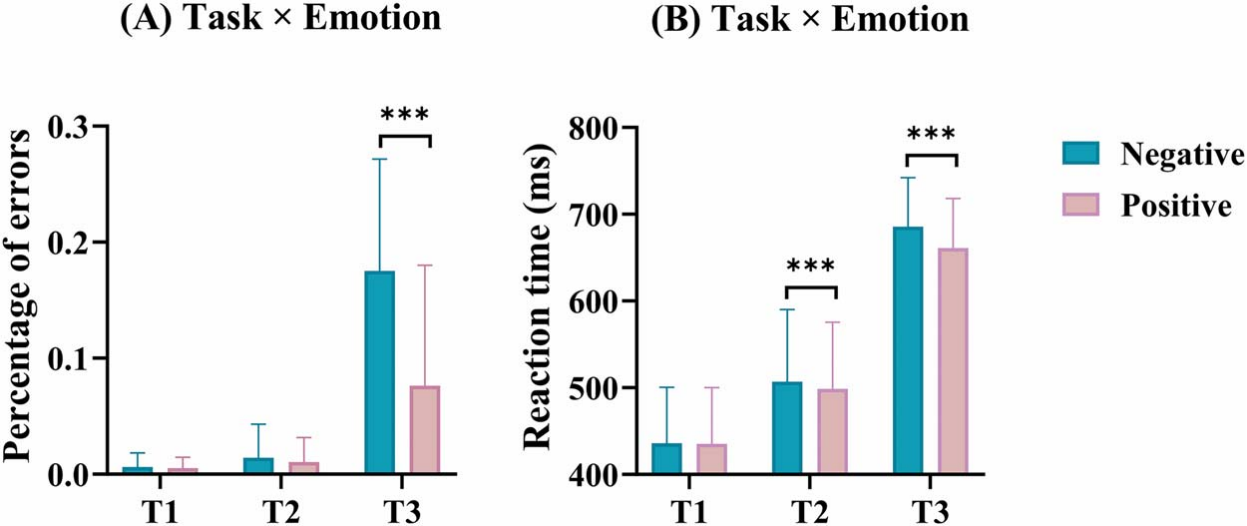
A) Percentage of errors for the two emotional valences in the three tasks; B) reaction time for the two emotional valences in the three tasks. ^*^P < 0.05, ^**^P < 0.01, ^***^P < 0.001.

The two-way repeated measures ANOVA of task × emotion on RT yielded statistically significant primary effects for both task (*F*_[2238]_ = 1,097.70, *P* < 0.001, η2 *P* = 0.90) and emotional condition (*F*_[1119]_ = 56.19, *P* < 0.001, η2 *P* = 0.32). The RT varied significantly across tasks, with Task 1 showing the fastest performance (435.58 ms ± 5.88) and Task 3 showing the slowest (673.09 ms ± 4.86). Regarding emotional effects, trials involving positive stimuli produced faster response (531.56 ms ± 5.30) compared to those with negative stimuli (542.69 ms ± 5.36; mean difference = −11.13, 95% CI = −14.07, −8.19). The analysis further identified a significant interaction between task and emotion types (*F*_[2238]_ = 24.92, *P* < 0.001, η2 *P* = 0.17). Follow-up comparisons with Bonferroni adjustment revealed that RT in Task 1 remained the fastest across all tasks regardless of the type of emotion (all *P* < 0.001), while Task 3 showed the slowest RTs (all *P* < 0.001), see Fig. 2. Both Tasks 2 and 3 showed emotional modulation, with faster response to positive stimuli (*P* < 0.001), see Fig. 2.

### PSD

**Fig. 3:**
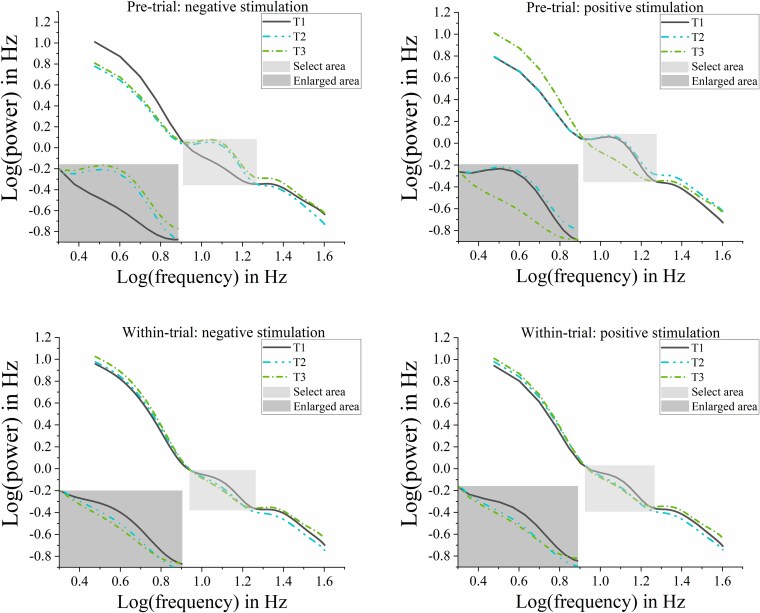
The grand-averaged (across all electrodes and participants) PSDs in log–log space. The upper panel compares PSDs between three different tasks with negative stimulation and positive stimulation during the pretrial phase (−1,000 to 0 ms prestimulus), while the lower panel shows corresponding spectra during within-trial phase (0 to 1,000 ms post-stimulus).

Figure 3 displays the PSDs in log–log coordinates, contrasting negative and positive stimulation conditions across the 3 to 40 Hz frequency range. The spectra represent grand averages across all electrodes and participants during both pretrial (−1,000 to 0 ms) and within-trial (0 to 1,000 ms) periods.

### Aperiodic exponent (brain-wide)

**Fig. 4:**
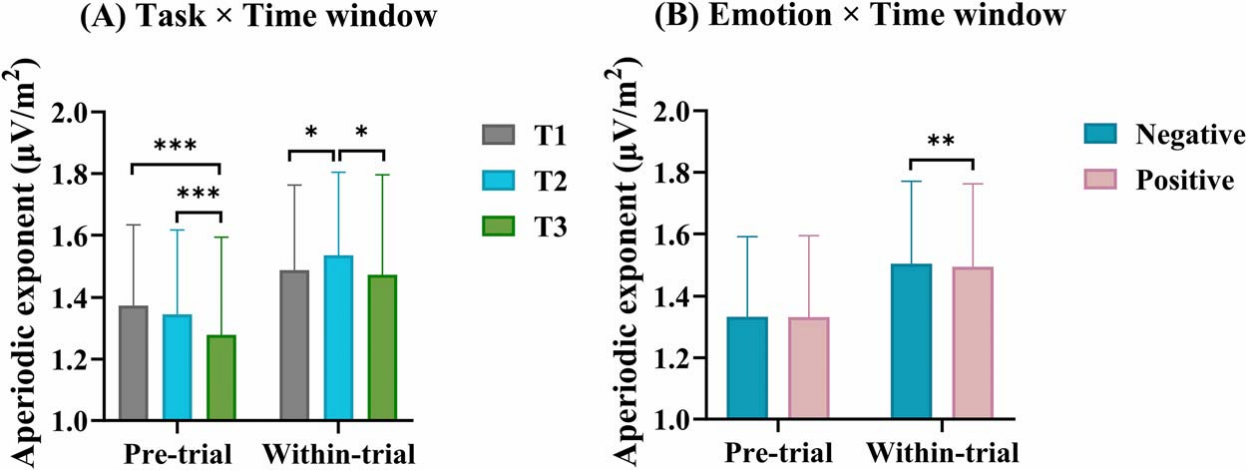
A) Aperiodic exponent for the three tasks in the pre-trial and within-trial periods B) aperiodic exponent for negative and positive emotions in the pretrial and within-trial periods. ^*^P < 0.05, ^**^P < 0.01, ^**^P < 0.001.

A three-way repeated measures ANOVA of task × emotion × time-window was performed. The results revealed two significant main effects. First, the main effect of task (*F*_[1119]_ = 8.30, *P* < 0.001, η2 *P* = 0.07, 95% CI = 0.156, 0.179) was significant implying that Task 3 (1.38 μV/m^2^ ± 0.03) has the lowest aperiodic exponent compared to Task 1 (1.43 μV/m^2^ ± 0.02, 95% CI = −0.096, −0.014) and Task 2 (1.44 μV/m^2^ ± 0.02, % CI = −0.107, −0.023). Second, the significant main effect of time-window (*F*_[1119]_ = 848.73, *P* < 0.001, η2 *P* = 0.88) showed that the aperiodic exponent was higher in the within-trial phase (1.50 μV/m^2^ ± 0.02, 95% CI = 0.156, 0.179) compared to the pretrial phase (1.33 μV/m^2^ ± 0.02). Moreover, there were two significant interaction effects of task × time-window (*F*_[2238]_ = 49.89, *p* = < 0.001, η2 *P* = 0.30), as illustrated in Fig. 4A and of emotion × time-window (*F*_[1119]_ = 7.07, *P* = 0.009, η2 *P* = 0.06), as depicted in Fig. 4B. Regarding the task × time-window interaction, in the pretrial, the aperiodic exponent in Task 3 (1.28 μV/m^2^ ± 0.03) was significantly lower than that in Task 1 (1.37 μV/m^2^ ± 0.02; *t* = 5.59, *P* < 0.001, *d* = − 0.10, 95% CI = −0.138, −0.053) and Task 2 (1.35 μV/m^2^ ± 0.03; *t* = 4.00, *P* < 0.001, *d* = −0.07, 95% CI = −0.110, −0.026). In the within-trial, instead, the aperiodic exponent in Task 2 (1.54 μV/m^2^ ± 0.03) was significantly higher than that in Task 1 (1.49 μV/m^2^ ± 0.03; *t* = 2.76, *P* < 0.05, *d* = 0.05, 95% CI = 0.006, 0.089) and in Task 3 (1.47 μV/m^2^ ± 0.03; *t* = 3.26, *P* < 0.01, *d* = 0.06, 95% CI = 0.016, 0.108), as shown in Fig. 4A. Regarding the emotion × time-window interaction, the aperiodic exponents in all three tasks was higher in the within-trial period compared to the pretrial period. Notably, in the pretrial, there was no significant difference in aperiodic exponents between the two types of emotions (1.33 μV/m^2^ ± 0.02 vs. 1.33 μV/m^2^ ± 0.02; *t* = 0.25, *P* = 0.84, *d* = 0.001, 95% CI = −0.007, 0.009), but, in the within-trial, the aperiodic exponent was significantly higher for negative emotions compared to positive emotions (1.50 μV/m^2^ ± 0.02 vs. 1.49 μV/m^2^ ± 0.03; *t* = 2.25, *P* < 0.01, *d* = 0.01, 95% CI = 0.002, 0.017), see Fig. 4B. Further, the aperiodic exponent was higher in the within-trial, compared to the pretrial period, irrespective of the type of emotions. No other significant interaction effect was found (*p*s > 0.05).

### Scalp topography and aperiodic exponent (electrode-specific)

**Fig. 5:**
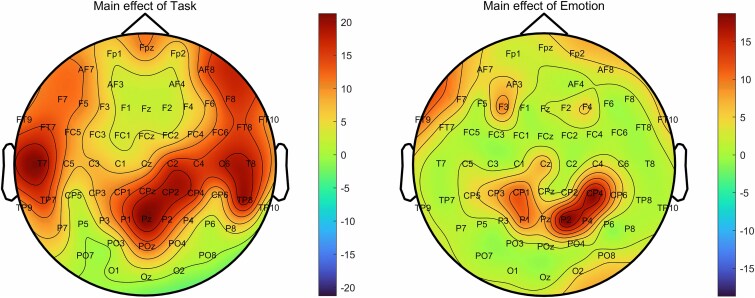
Scalp distributions of the aperiodic exponent. The figures show electrode sites with a significant main effect of task and emotion. Colors indicate cluster-level summed F-values.

To identify the electrodes contributing to the observed aperiodic exponent effects, we then performed a cluster-based permutation test on within-trial period. The permutation result of task and emotion on within-trial periods revealed significant main effects of task across multiple brain regions including frontal, central, temporal, posterior, and occipital areas: Cluster 1 (0–7, Fp1, Fp2, F3, F4, C3, C4, *F*_[1119]_ = 15.29, *P* < 0.01, η2 *P* = 0.27); Cluster 2 (10–15, F7, F8, T7, T8, P7, P8, *F*_[1119]_ = 20.89, *P* < 0.01, η2 *P* = 0.27); Cluster 3 (17, 18, Cz, Pz, *F*_[1119]_ = 21.29, *P* < 0.01, η2 *P* = 0.75); Cluster 4 (20–25, FC1, FC2, CP1, CP2, FC5, FC6, *F*_[1119]_ = 19.22, *P* < 0.01, η2 *P* = 0.31); Cluster 5 (27, 28, CP6, TP9, *F*_[1119]_ = 8.37, *P* < 0.05, η2 *P* = 0.56); Cluster 6 (33–42, C1, C2, P1, P2, AF3, AF4, FC3, FC4, CP3, CP4, *F*_[1119]_ = 16.36, *P* < 0.01, η2 *P* = 0.17); Cluster 7 (44–48, F5, F6, C5, C6, *F*_[1119]_ = 17.77, *P* < 0.01, η2 *P* = 0.35); Cluster 8 (51–56, AF7, AF8, FT7, FT8, TP7, TP8, PO7, *F*_[1119]_ = 19.46, *P* < 0.01, η2 *P* = 0.22); Cluster 9 (59–63, FT9, FT10, Fpz, CPz, *F*_[1119]_ = 17.08, *P* < 0.01, η2 *P* = 0.32), and main effect of emotion across areas including Cluster 1 (1, 2, 3, *F*_[1119]_ = 9.24, *P* < 0.05, η2 *P* = 0.47), and Cluster 2 (35, 36, 37, *F*_[1119]_ = 18.71, *P* < 0.05, η2 *P* = 0.54), and Cluster 3 (41, 42, *F*_[1119]_ = 18.14, *P* < 0.05, η2 *P* = 0.72). No interaction effect was found. The spatial distribution of aperiodic exponents across the scalp is illustrated in Fig. 5.

Electrodes exhibiting t values greater than 6 were identified, indicating the most substantial effects in these brain regions. Aperiodic exponents at T7, Pz, CP2, exhibited the most significant effects, warranting a focused statistical analysis on these three electrodes. After averaging the aperiodic exponents of electrodes T7, Pz and CP2 for each participant and each condition, we performed a 3 (Task 1 vs. Task 2. Vs. Task 3) × 2 (emotion: positive vs. negative) repeated measures ANOVA. The results resembled the brain-wide analysis showing a main effect of emotion, (*F*_[1119]_ = 6.25, *P* < 0.05, η2 *P* = 0.05), and task, (*F*_[2238]_ = 18.45, *P* < 0.001, η2 *P* = 0.13).

### Resting state

We tested whether the aperiodic exponent at resting-state would predict individual differences in emotional responsiveness. However, Pearson correlation (brain-wide) analysis showed that the baseline resting-state aperiodic exponent was not significantly related to Δ-emotion (r = −0.17, *P* > 0.05).

### BIG 5

Finally, a descriptive analysis of the BIG 5 questionnaire yielded the following results across personality dimensions: Neuroticism (Mean = 26.87, SD = 6.74), Conscientiousness (Mean = 33.76, SD = 5.41), Agreeableness (Mean = 35.87, SD = 5.16), Openness (Mean = 32.64, SD = 6.46), and Extraversion (Mean = 29.46, SD = 7.46). All dimensions demonstrated acceptable univariate normality, with absolute values of skewness < 0.6 and kurtosis < 0.3, supporting the appropriateness of subsequent parametric statistical approaches (Byrne 2016).

**Fig. 6:**
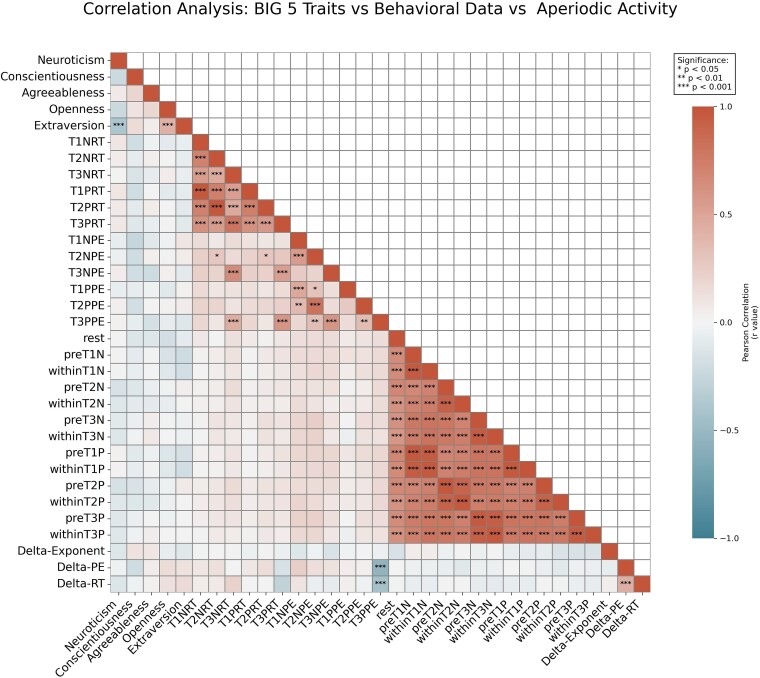
After Bonferroni correction, correlation matrix relating BIG 5 scales, behavioral measures, and aperiodic exponents. Labels: T1/T2/T3 (Tasks 1 to 3), N/P (negative/positive valence), RT/PE (reaction time/percentage of error), pre/within (pretrial, within-trial), Delta-exponent (negative FOOOF across all tasks in within-trial minus positive FOOOF across all tasks in within-trial), Delta-PE (negative PE across all tasks minus positive PE across all tasks), Delta-RT (negative RT across all tasks minus positive RT across all tasks). ^***^P < 0.001.

An exploratory correlational analysis showed that, after statistical correction (see Fig. 6), no significant correlations were observed between individual variability in aperiodic EEG activity and emotion. This suggests that, within the current sample and tasks, aperiodic cortical dynamics do not appear to be systematically related to differences in emotional reactivity or regulation, highlighting the need for further investigation in larger or more targeted studies.

## Discussion

The goal of this study was 2-fold. First, we aimed to test empirical implications of the claim that metacontrol and emotion are tightly related (Dreisbach and Goschke 2004) or even strongly overlapping with respect to their underlying neural mechanisms (Hommel 2019, 2025). If they would be, we reasoned, the previously reported relationship between metacontrol biases towards persistence or flexibility on the one hand and increases or decreases of the aperiodic exponent (Donoghue et al. 2020) on the other (Gao et al. 2024, 2025; Pi et al. 2024, 2025; Yan et al. 2024, 2025) would suggest that positive emotions, which have been found to foster flexibility and impair persistence, should come with a lower aperiodic exponent than negative emotions, which were found to support persistence, but not flexibility (see Hommel and Colzato 2017). Our findings fully confirmed this prediction, which is in line with the assumption that metacontrol and emotion are interconnected, if not partly identical.

Our findings are consistent with a constructivist account of human emotion, as suggested by Barrett (2017). The basic idea is that emotions are not necessarily a natural kind but constructed on the basis of self-perception. This perception is assumed to be grounded in more or less autonomous neural mechanisms, including various kinds of executive functions, so that the phenomenal experience of emotions can be taken to represent how particular regulatory functions “feel”. More specifically and with respect to the present study, shifting metacontrol towards the persistence pole might be accompanied by the phenomenal experience of negative affect, whereas shifting metacontrol towards flexibility might come with the experience of positive affect. This would not require any causal impact of felt emotion on cognitive-control style, but it would suggest that metacontrol shifts are sharing some neural basis with the generation of emotional feelings. As discussed in the introduction already, neurochemical assumptions in both emotion research and approaches to metacontrol do indeed suggest such a shared basis, for which dopamine, serotonin, and the interaction between prefrontal areas and the striatum are likely to play a major role (Cools et al. 2008; Durstewitz and Seamans 2008; Hommel and Colzato 2017). A strong practical benefit of such a mechanistic overlap would be that the (reportable) affective states of people might be used as a cue indicating the present (unreportable) metacontrol bias (Hommel 2019).

Our second aim was motivated by the ongoing discussion regarding the degree to which emotional stimuli are affecting human decision-making in an automatic fashion (Zajonc 1980; Barrett 2017). If they would do, their effects should not depend on the degree to which the emotional content of such stimuli is relevant or irrelevant to the present task and the current goals of the agent. To test whether they do depend on the task context, we systematically manipulated the task-relevance of the same emotional stimuli in the three tasks of the present study. The results showed a dissociation between overt behavior and aperiodic exponents. In both error rates and reaction times, the task context mattered a lot: error rates were modulated by emotion only in Task 3, where the emotional content was task-relevant, and the degree to which emotion modulated reaction times increased with the degree of task-relevance. In contrast, aperiodic exponents showed strong effects of emotion but no sign of modulation by task. How can this discrepancy be explained?

For one, there are good reasons not to over-interpret the findings for error rates and reaction times. The result patterns show some indication of floor effects, especially in the error rates—where performance was way too accurate to allow any modulation by emotion. Similarly in reaction times, where the numerically larger emotion effects are associated with longer reaction times overall. Given that higher effect sizes in reaction times can be expected with higher levels of reaction time for purely statistical reasons, it is not entirely clear whether the observed interaction reflects a real effect. For another, emotions are likely to affect performance in various ways, and not all of them need to be related to metacontrol. More specifically, positive affect is likely to have motivational effects, which may account for the pattern observed in overt behavior. Accordingly, we do not consider the discrepancy between the results obtained for overt behavior on the one hand and in aperiodic exponents on the other as a contradiction but, rather, assume that they reflect different kinds of effects. Moreover, a recent study has shown that stimulus-induced shifts of metacontrol do not necessarily have an impact on the present trial but affect the processing style in the next trial (Jia et al. 2024). This can be taken to suggest that metacontrol shifts are relatively slow as compared to task-specific cognitive operations, which in turn suggests that discrepancies between overt behavior changes in aperiodic activity in the same trial are indeed to be expected.

Nevertheless, the fact that task context did matter for overt behavior measures but not for the aperiodic exponent suggests an interesting perspective. Based on the overt measures only, our study would suggest that task conditions make a difference for the degree to which emotional stimuli can affect behavior. This difference can apparently be very substantial, as indicated by the significant interactions in both error rates and reaction times. From a truly and fully automatic process, one would have expected a stronger impact of the emotional valence and a lesser effect of the task. Hence, from a behavioral perspective, our findings do not support the assumption of full automaticity. However, based on our findings for aperiodic exponents, the assumption of full automaticity is very plausible, as task did not matter at all. Taken together, this discrepancy seems to suggest that the question whether the impact of a particular stimulus or stimulus type is or is not automatic might be too simplistic without specifying the process one believes to be affected. In other words, the same stimulus might have some effects on human cognition and behavior that are automatic and other effects that are not. If so, the impact of emotional content, and positive and negative valence in particular, on metacontrol seems to be more automatic than its impact on overt behavior. Under the hypothesis that metacontrol and emotion share a substantial degree of their neural and neurochemical underpinnings, this seems to make perfect sense. Indeed, if some of the processes underlying metacontrol and emotion are identical (Hommel 2019), automaticity is a necessary consequence.

A critical feature of our design is that Task 3 differs from Tasks 1 and 2, requiring explicit classification of emotional valence rather than gap-direction judgments. This distinction was intentional, as it allows investigation of controlled, task-relevant emotional processing in contrast to the task-irrelevant manipulations of the first two tasks. While Task 3 is cognitively more demanding and may engage additional attentional and arousal mechanisms, our statistical approach accounts for these differences: behavioral and aperiodic EEG data were analyzed using separate repeated-measures ANOVAs with task as a within-subject factor, allowing us to examine task-specific effects as well as interactions with emotion type. The calculation of pre-trial and within-trial aperiodic exponents ensures that task-specific cognitive activity is captured independently of baseline differences. Moreover, all post hoc and cluster-based analyses were performed within tasks or task-relevant clusters, preserving the interpretability of results despite differences in task demands. Therefore, while Task 3 differs from Tasks 1 and 2 by design, its inclusion is methodologically justified and supports the study’s goal of dissociating automatic vs. controlled emotional processing.

While the present study interprets variations in aperiodic EEG activity as reflecting emotion-related neural modulation, it is important to note that this interpretation is based on theoretical and empirical evidence rather than direct behavioral verification of emotional states. A prior study has shown that the aperiodic exponent reflects cortical E/I balance, which is sensitive to affective arousal (Borah et al. 2025). In our event-related design, the aperiodic changes observed likely reflect transient neural responses elicited by the emotional stimuli, rather than a sustained mood state. Although a blocked design with post-block emotion ratings could more directly capture subjective affect, such an approach would increase habituation and expectancy effects, which are particularly problematic in tasks where emotion is behaviorally relevant. Even though the IAPS is a reliable tool for emotion elicitation and demonstrates robust cross-cultural applicability (Branco et al. 2023), future studies should incorporate post-task valence or arousal ratings, or complementary physiological measures, to further validate the relationship between aperiodic activity and experienced emotion.

Taken altogether, our study shows that emotional stimuli have an impact on the aperiodic exponent, in the sense that positive stimuli induce lower values of the exponent, implying more cortical noise, than negative stimuli. This observation demonstrates a strong, mechanistically traceable link between emotional content and aperiodic activity in general and between emotion and metacontrol in particular. Moreover, it points to aperiodic activity and, from a functional point of view, cortical noise as the common currency of emotion and control. We also found that the task context and the task-relevance of the emotional stimuli in particular have a substantial impact on overt behavior but not on the aperiodic exponent. This pattern is not consistent with the assumption of a fully automatic impact of emotional stimuli on behavior but suggests that the impact of such stimuli on metacontrol shifts might indeed be automatic.

## Data Availability

All data can be obtained from Jing Fan (lyra800730@163.com) upon reasonable request.
